# Narcolepsy Beyond Medication: A Scoping Review of Psychological and Behavioral Interventions for Patients with Narcolepsy

**DOI:** 10.3390/jcm14082608

**Published:** 2025-04-10

**Authors:** Giorgia Varallo, Alessandro Musetti, Maria Filosa, Giada Rapelli, Fabio Pizza, Giuseppe Plazzi, Christian Franceschini

**Affiliations:** 1 Department of Biomedical, Metabolic and Neural Sciences, University of Modena and Reggio Emilia, 41125 Modena, Italy; g.varallo@unimore.it (G.V.); giuseppe.plazzi@isnb.it (G.P.); 2 Department of Humanities, Social Sciences and Cultural Industries, University of Parma, 43121 Parma, Italy; alessandro.musetti@unipr.it (A.M.); maria.filosa@unipr.it (M.F.); 3 Department of Psychology, University of Bologna, 40127 Bologna, Italy; giada.rapelli@unibo.it; 4 IRCCS Istituto delle Scienze Neurologiche di Bologna, 40139 Bologna, Italy; fabio.pizza@unibo.it; 5 Department of Biomedical and Neuromotor Sciences, Alma Mater Studiorum, University of Bologna, 40127 Bologna, Italy; 6 Department of Medicine and Surgery, University of Parma, Via Gramsci, 14, 43126 Parma, Italy

**Keywords:** behavioral sleep medicine, clinical psychology, cognitive and behavioral therapy, psychological intervention, narcolepsy

## Abstract

**Objectives**: The present scoping review aims to map the existing evidence on psychological and behavioral interventions targeting patients with narcolepsy type 1 and type 2. **Methods**: A literature search was performed using the databases Scopus, PubMed, and PsycINFO. Studies were included if they (1) employed randomized controlled trials, non-randomized trials, or quasi-experimental studies; (2) were published in English; (3) were published in peer-reviewed journals; (4) examined the impact of psychological interventions on psychopathological (primary outcomes) and narcolepsy-related symptoms (secondary outcomes); and (5) involved patients with a diagnosis of narcolepsy using recognized diagnostic criteria regardless of whether they were receiving pharmacological treatment or were untreated. No restrictions were imposed on the publication date to comprehensively map the available evidence. Data were extracted to address the review aims and presented as a narrative synthesis. **Results**: The database search yielded six studies. Treatment options for individuals with narcolepsy encompass psychological and behavioral interventions, such as telehealth interventions, meditation/relaxation therapy, and scheduled napping. The primary outcomes were daytime sleepiness, wakefulness maintenance, sleep attacks, the severity of symptoms of narcolepsy, sleep paralysis episodes, depression, and psychological functioning. The secondary outcomes were sleep-problem-related quality of life, sleep inertia, and sleep quality. The psychological and behavioral interventions exhibited variability in terms of the intervention type, personnel involved, number of sessions, and duration. Most of the contributions also lack details regarding the training of professionals and the specifics of the interventions. Additionally, the evidence quality was deemed low based on the Crowe Critical Appraisal Tool. **Conclusions**: Although the importance of nonpharmacological approaches is well recognized, there is limited evidence to support the efficacy of psychological and behavioral interventions in narcolepsy. This is further complicated by the wide range of psychological and behavioral interventions available.

## 1. Introduction

Narcolepsy is a sleep disorder marked by symptoms such as excessive daytime sleepiness, sleep paralysis, cataplexy, and hypnagogic hallucinations [1]. According to the third version of the International Classification of Sleep Disorders by the American Academy of Sleep Medicine [2], narcolepsy is classified into two subtypes: narcolepsy type 1 (NT1) and narcolepsy type 2 (NT2). NT1 presents cataplexy and significantly reduced hypocretin levels in the cerebrospinal fluid, due to the selective loss of hypocretin-producing neurons in the hypothalamus [3,4]. 

In contrast, NT2 is characterized by normal cerebrospinal fluid hypocretin levels and the absence of cataplexy. The onset typically occurs during adolescence or early adulthood, with a bimodal peak at approximately 15 and 35 years of age [5]. The prevalence of narcolepsy ranges from 25 to 50 cases per 100,000 people [6]. In Italy, the estimated prevalence is approximately 40 cases per 100,000, with significant underdiagnosis suggesting that at least 24,000 individuals remain undiagnosed [7].

Pharmacological treatments, including stimulants and antidepressants, remain the primary approach for managing narcolepsy [8]. However, many patients with narcolepsy, despite treatment, experience debilitating symptoms and impaired quality of life [9]. While pharmacotherapy effectively reduces excessive daytime sleepiness and cataplexy, patients frequently report unmet needs, particularly in the psychosocial and emotional domains. Indeed, the impact of narcolepsy on daily functioning, emotional well-being, and social relationships is profound and multifaceted [10,11,12,13]. This burden extends to the families of patients, such as spouses and parents in the case of pediatric or adolescent patients [14,15]. The association between narcolepsy and psychiatric conditions is particularly significant. Recent systematic reviews have estimated that depressive symptoms affect nearly one-third of patients with narcolepsy [16], with other studies reporting persistent moderate to severe depressive symptomatology in approximately 25–29% of cases [17]. Moreover, Fortuyn et al. [18] observed that anxiety predominantly outweighs depression in the psychiatric profile of narcolepsy patients, with a notable prevalence of panic attacks and social phobia in comparison to the general population.

Non-pharmacological interventions, including psychological and behavioral strategies, have been proposed as adjuncts to pharmacotherapy in narcolepsy management. These interventions aim to enhance symptom control, mitigate psychiatric comorbidities, and improve overall functioning and quality of life [6,19,20]. Treatment guidelines from organizations such as the American Academy of Sleep Medicine [21] and the European Federation of Neurological Societies [22] endorse psychological and behavioral strategies, including scheduled naps, cognitive behavioral approaches, and psychosocial support, as valuable components of comprehensive care. Despite these recommendations, the scope of evidence for psychological and behavioral interventions remains unclear.

This scoping review aims to map the existing literature and provide a summary of psychological and behavioral interventions aimed at (1) reducing psychopathological symptoms such as depressive and anxiety symptomatology and (2) reducing narcolepsy-related symptoms, including daytime sleepiness, sleep paralysis, and cataplexy. The review will examine the following aspects: (1) the characteristics of the interventions, (2) the specific strategies employed, (3) the clinical approach underpinning the interventions, (4) the providers of these interventions, and (5) the reported short-term and long-term effects on primary and secondary endpoints.

## 2. Materials and Methods

Our scoping review followed the methodological framework developed by Arksey and O’Malley [23], which includes (1) research question formulation, (2) identification of relevant studies, (3) selection of studies, (4) data extraction, and (5) synthesis and reporting of results. Additionally, the review adhered to the Preferred Reporting Items for Systematic Reviews and Meta-Analyses Extension for Scoping Reviews guidelines [24] to ensure transparency and methodological rigor.

### 2.1. Inclusion and Exclusion Criteria

This review included only original research articles that met the following criteria: (1) employed randomized controlled trials (RCTs), non-randomized trials, or quasi-experimental studies; (2) were published in English; (3) were published in peer-reviewed journals; (4) evaluated the effect of psychological and behavioral interventions on psychopathological (primary outcomes) and narcolepsy-related symptoms (secondary outcomes); (5) involved patients with a diagnosis of narcolepsy using recognized diagnostic criteria regardless of whether they received pharmacological treatment or were untreated.

### 2.2. Search Strategy

The search was conducted across the databases PubMed, Scopus, and PsycINFO from July to October 2024. The search strategy was developed according to the Patient/Population, Intervention, Comparison, and Outcome (PICO) framework. It combined relevant keywords and Medical Subject Heading (MESH) terms, resulting in the following search string: (“narcolep*”) AND (“psychotherapy” OR “psych*” OR “counsel*” OR “peer support” OR “parent training” OR “therap*” OR “self-management” OR “coping” OR “behavioral therapy” OR “psychosocial” OR “mindful*” OR “treatment” OR “intervention*” OR “nap*”). Boolean operators and truncation techniques were applied to account for variations in terminology and to ensure inclusivity. Synonyms and related terms were included to enhance inclusivity, particularly for intervention-related keywords. Filters such as publication year and study type were not applied. Search syntax was adapted as necessary for each database to align with its indexing structure.

### 2.3. Data Extraction and Quality Assessment

After removing duplicates, two researchers (M.F. and G.V.) independently screened for eligibility the titles and abstracts of identified studies. Full-text articles were then assessed. Disagreements between researchers were resolved through consensus. A manual search of the reference lists from all included studies was conducted but yielded no additional included articles. Data extraction and quality assessment were performed using the Crowe Critical Appraisal Tool (CCAT, v1.4), applied independently by the two blinded authors. The CCAT evaluates eight distinct domains: preliminaries, introduction, study design, sampling, data collection, ethics, results, and discussion. Each domain is scored on a scale of 0–5, providing a percentage score. This approach allows for a transparent assessment of study quality, considering both the overall score and individual domain scores to identify methodological strengths and weaknesses across included studies.

## 3. Results

### 3.1. Study Selection

**Figure 1:**
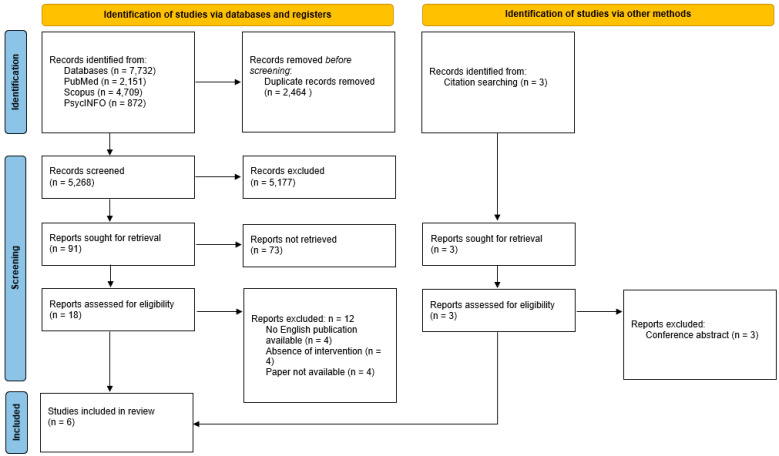
PRISMA flowchart displaying the number of studies identified, screened, and included/excluded, with the reasons for exclusion.

A search of electronic databases provided a total of 7731 records. After the removal of duplicates, 5268 records remained. The abstracts were examined, leading to the exclusion of 5178 articles based on predefined inclusion criteria. A subset of 90 records was selected for further evaluation, and 18 articles underwent comprehensive assessment through a full-text review. Eventually, 12 records were excluded during this evaluation phase. Consequently, six records were considered suitable for inclusion [25,26,27,28,29,30]. The study selection process is depicted in Figure 1.

### 3.2. Study Quality

The quality assessment scores for each study are reported in Table 1. Among the selected studies, the design, sampling, results, and discussion categories generally received lower quality assessment scores. Several factors contributed to low scores, including the absence of a control group, a non-randomized study design, a small sample size, and missing flow of participants through each stage of research such as an appropriate description of demographic and other characteristics of participants. Furthermore, methods for managing missing data were not identified in the studies reviewed, and none of the studies selected compare the intervention group with an active control group receiving a gold-standard intervention. Two studies received the label NA (not applicable) in the ethical category. This was due to a complete absence of relevant information concerning ethical approval, funding sources, informed consent, and other essential ethical elements in these studies.

### 3.3. Description of Participants

The selected studies included a total of 158 adult participants diagnosed with narcolepsy including both female and male patients (sex assigned at birth), with females making up the majority (70%). In one study, specific sex distribution data were not reported [25]. The sample size varied from a minimum of 8 patients [26] to a maximum of 60 patients [27]. The mean age of participants was 38.61 years. One study included participants diagnosed with NT1 [26]. Two studies involved patients with both NT1 and NT2 [25,27], and another focused on patients suffering from central disorders of hypersomnolence, which is the broad category of sleep disorders in which narcolepsy type 1 and 2 are included [28]. Lastly, two studies referred to participants as “narcoleptic” without specifying the subtype [29,30]. 

### 3.4. Study Characteristics

The studies included are described in Table 2 and Table 3. The selected studies were published from 1993 to 2024. Four studies were conducted in the USA [26,27,28,29], one in Italy [25], and one did not report the country location [30]. Two studies employed a pretest–posttest design [26,30], one study was a non-randomized control trial [25], one study implemented an RCT design [29], and one study employed a feasibility design [27]. The intervention duration ranged from 8 days [26] to 12 weeks [27].

### 3.5. Description of Interventions

#### 3.5.1. Intervention Group

The interventions implemented in the selected studies are outlined in Table 4. The delivery methods varied. In three studies evaluating nap therapy, participants received in-person instructions regarding their napping schedules; however, the intervention itself (i.e., the nap schedule) was carried out by participants at home [29,30] and in the experimental setting [26]. One study adopted a hybrid approach, combining in-person sessions with an online component to deliver instructions on the relaxation-based intervention, which was subsequently implemented independently by participants [25]. Finally, the remaining two studies exclusively conducted the intervention in a fully remote format delivered by a therapist [27,28]. The number of intervention sessions ranged from 6 [28] to 12 [26,27]. However, three studies did not provide explicit information on the total number of single sessions included in the interventions [25,29,30]. Provider expertise varied significantly. Only one intervention was delivered by a clinical psychologist [28]. In another study, the provider was a polysomnographic technologist [30]. One study reported that the intervention was delivered by a licensed health professional trained in mindfulness [27], and in three studies, the provider’s background was not reported [25,26,29].

The proposed interventions implemented several psychological and behavioral theoretical approaches. Jalal et al. [25] evaluated a cognitive and behavioral psychological intervention targeting sleep paralysis. The protocol implemented four specific techniques: cognitive reframing to reinterpret the episode, emotional distancing to reduce catastrophizing, inward-focused attention meditation on positive emotional objects, and muscle relaxation with non-judgmental acceptance of physical symptoms. The sessions were brief, conducted twice weekly for 15 min.

Ong et al. [28] developed and implemented cognitive behavioral therapy for hypersomnia (CBT-H) in individual and group formats. The intervention modules addressed various aspects, including central disorders of hypersomnolence (education, identity and self-image, daily routines, coping skills, emotion regulation, and social support). While the intervention content was detailed, specific psychological strategies were not explicitly described.

Mundt et al. [27] assessed the feasibility and efficacy of a mindfulness-based intervention adapted for narcolepsy. Sixty adults were randomized into three groups with intervention durations of 4, 8, or 12 weeks. The program, adapted from mindfulness-based stress reduction, included mindfulness practices such as body scans, sitting and walking meditation, and yoga, with adjustments to accommodate alertness challenges associated with narcolepsy.

Three studies examined interventions based on a behavioral approach and centered on the role of scheduled naps [26,29,30]. Rogers and Aldrich [30] proposed a program with three regularly scheduled 15 min naps during the day. In another study [26] proposed three nap schedules: (1) a single consolidated nocturnal period without any naps; (2) a long nap schedule; and (3) a multiple short nap condition consisting of five evenly spaced naps spread throughout the day. Finally, Rogers and colleagues [29] proposed three different sleep schedules: (1) two 15 min naps per day; (2) a regular sleep schedule; or (3) a combination of scheduled naps and regular sleep schedules.

#### 3.5.2. Control Group

Two out of the six studies compared the intervention group with a control group [25,29]. Specifically, Jalal et al. [25] used deep breathing techniques, and Rogers et al. [29] used two regularly scheduled 15 min naps per day (Control Group-1) and a regular schedule for arising and retiring each day (Control Group-2). The last three studies did not include a control group [26,27,30] comparisons between different interventions. In the study by Ong et al. [28], there were two intervention groups: individual format (Intervention group-1) vs. group format (Intervention Group-2). Mundt et al. compared three intervention formats of varying durations: brief (4 weeks), standard (8 weeks), extended (12 weeks) [27]. Similarly, both the studies by Mullington and Broughton [26], and Rogers and Andrich [30] involved a comparison between different nap protocols.

### 3.6. Effects of the Intervention Across Time Points

#### 3.6.1. Primary Outcomes

Three studies investigated daytime sleepiness as a primary outcome [26,28,29] using different measures. Mullington et al. [26] opted for a series of performance measures, such as descending subtraction test, a grammatical transformation or logical reasoning test, four-choice reaction time, a grip strength test, a measure of oral temperature, and a number of additional subjective evaluation questions. Ong et al. [28] employed the Epworth Sleepiness Scale. Finally, Rogers et al. [29] measured daytime sleepiness using 24 h ambulatory polysomnographic monitoring.

The heterogeneity of measures highlights the complexity of synthesizing results, necessitating separate discussions of each study’s findings. Mullington and Broughton [26] demonstrated the effectiveness of napping strategies in reducing daytime sleepiness in NT1 patients after eight days. However, no differences were found between intervention groups comparing long and multiple short nap conditions (five equidistant naps throughout the day). Ong et al. [28] observed a significant reduction in daytime sleepiness measured by the Epworth Sleepiness Scale at the six-month follow-up in the total sample, but no differences among groups. Finally, the study by Rogers et al. [29] showed a significant decrease in daytime sleepiness at the two-week follow-up in the total sample, with the combination therapy showing a greater reduction in daytime sleep duration compared to the control groups.

Similarly to daytime sleepiness, daytime alertness and sleep attacks were measured as primary outcomes in the study by Rogers and Andrich [30]. The daytime alertness was measured using the Maintenance of Wakefulness Test. Sleep attacks were measured with self-reported sleep diaries. They found that sleep latency significantly increased at one-month follow-up; however, there was no significant reduction in the frequency of sleep attacks.

In two studies [29,30], the severity of narcolepsy symptoms was measured as the primary outcome using the self-report Narcolepsy Symptom Status Questionnaire. Both studies reported no significant change in Narcolepsy Symptom Status Questionnaire scores across time points. In one study [25], sleep paralysis was measured as the primary outcome in terms of the number of days, number of episodes per day, and duration of sleep paralysis in the last month using the self-report Sleep Paralysis Experiences and Phenomenology Questionnaire. The results showed a significant reduction in the number of days sleep paralysis occurred and a significant reduction in the total number of episodes during the last month at the two-month follow-up. A reduction in the duration of episodes is not significant at two-month follow-ups.

In the study by Mundt et al., aimed at evaluating the feasibility of a mindfulness-based intervention, the primary outcomes revealed that the benchmarks for attendance, meditation practice, and data completeness were achieved in 71.7%, 61.7%, and 78.3% of cases, respectively [27]. Participants assigned to the brief and extended intervention conditions demonstrated greater compliance with the prescribed benchmarks relative to those in the standard intervention condition.

#### 3.6.2. Secondary Outcomes

Only the study by Ong et al. [28] also reported secondary outcomes. In particular, sleep-problem-related quality of life measured with the Functional Outcomes of Sleep Questionnaire, sleep inertia measured with Sleep Inertia Questionnaire, and sleep quality measured with Restorative Sleep Questionnaire were assessed. The results for all secondary outcomes showed no significant differences in the six-week follow-up.

In the study conducted by Mundt et al. [27], all versions of the intervention (brief, standard, and extended) improved mindfulness and self-compassion, with medium to large effect sizes. Extended mindfulness-based intervention had the most comprehensive impact, improving anxiety, depression, social and cognitive functioning, fatigue, and hypersomnia symptoms, while also reducing excessive daytime sleepiness. Standard mindfulness-based intervention had moderate benefits, while the brief mindfulness-based intervention had the least impact but still demonstrated improvements in emotional self-efficacy and fatigue.

## 4. Discussion

The primary purpose of this scoping review was to conduct an examination of existing evidence on psychological interventions aimed at reducing (i) psychopathological symptoms, such as anxiety and depression, and (ii) symptoms directly related to narcolepsy, such as daytime sleepiness, sleep paralysis, and cataplexy. The lack of studies evaluating the effectiveness of psychological and behavioral interventions in promoting psychological well-being and symptom management is the first striking finding. In fact, only six studies met our inclusion criteria and were included.

### 4.1. The Effect of Napping on Daytime Sleepiness

The three studies that investigated the effect of napping on daytime sleepiness produced consistent results [26,29,30]. A significant reduction in daytime sleepiness was consistently observed regardless of the specific napping schedule implemented in each study (combination of scheduled naps and regular bedtimes vs. three regularly scheduled naps vs. three sleep schedule protocols: no nap, long nap schedule, and multiple short nap schedule). This finding might suggest that napping remains effective regardless of the schedule used, indicating its potential flexibility and adaptability. Such clinical implications are beneficial because they may imply that the napping regimen can be tailored to individual needs and preferences, allowing for a more personalized approach to managing daytime sleepiness in patients rather than a strict and predetermined one. Two of these studies [29,30] found that individuals with severe daytime sleepiness improved significantly when they incorporated regular sleep periods into their daily schedules. However, individuals who exhibited only moderate sleepiness or who were able to maintain their alertness adequately did not experience significant benefits from such scheduled sleep periods. These results shed light on the potential benefits of individualized sleep interventions based on the severity of daytime sleepiness individuals experience.

These consistent findings are noteworthy, especially given the differences in daytime sleepiness assessments across studies. In fact, the included studies evaluated sleepiness using both self-reported measures like the Stanford Sleepiness Scale and objective measures like polysomnographic monitoring. Future studies could benefit from a combination of self-report and objective measures to gain a more comprehensive understanding of the impact of napping on daytime sleepiness. This method would facilitate comparison and improve the overall interpretation of the results.

However, all three of these studies exhibit several shared limitations. Firstly, these studies, mostly published in the 1990s and early 2000s, cannot be considered current evidence. They suffer from small sample sizes and fail to assess long-term effects. Furthermore, only one of these three studies included a control group that did not receive any napping intervention.

### 4.2. The Effect of Psychological Intervention on Daytime Sleepiness

Ong et al. developed a targeted CBT-H intervention to address central disorders of hypersomnia [28]. Remarkably, the intervention demonstrated potential in reducing excessive daytime sleepiness, thereby suggesting its effectiveness in managing this symptom.

Notably, the CBT-H protocol includes modules that target both behavioral and cognitive aspects of the disorder. Among these modules, specific attention is given to optimizing sleep hygiene and implementing scheduled naps. Likely, the inclusion of behavioral modules focusing on sleep hygiene and scheduled naps contributed to this favorable outcome. Due to the intervention’s complexity and multifaceted nature, however, it is unclear which specific component is responsible for the observed effect. The study has several limitations, including the lack of a control group and the small sample size. In addition, the assignment of treatment format as opposed to randomization may introduce selection bias. In this study, excessive daytime sleepiness was evaluated solely based on self-reported measures. However, for future investigations, it could be suggested to adopt a randomized controlled trial design, employ a larger sample size, and utilize both self-reported and objective measures to assess excessive daytime sleepiness more comprehensively. In addition, in order to assess whether CBT-H is more effective in reducing daytime sleepiness than napping alone, it would be useful to compare these two treatments.

### 4.3. The Effect of Napping Intervention on Symptom Severity

Two studies [29,30] evaluated the effect of a napping intervention on the severity of narcolepsy symptoms evaluated with the self-administered Narcolepsy Symptom Status Questionnaire that assessed excessive daytime sleepiness, sudden sleep attacks, cataplexy (sudden loss of muscle tone), hypnagogic hallucinations (visual or auditory sensations before falling asleep), and sleep paralysis. However, in both studies, consistent findings were observed, with no noteworthy modifications in Narcolepsy Symptom Status Questionnaire scores observed across various time points.

### 4.4. Sleep Paralysis

One study evaluated the efficacy of a muscle relaxation intervention for reducing SP [25]. The implementation of the intervention over an 8-week period led to a remarkable reduction in the occurrence of SP events. Considering that SP is a distressing symptom often associated with fear and terror [25,31] and that muscle relaxation interventions are a simple and self-managed technique applicable in a home environment, additional research is required to validate and confirm these findings due to the small sample size and non-randomized design. Furthermore, the study only looked at short-term effects; therefore, the long-term effects of muscle relaxation on SP are unknown.

### 4.5. Other Sleep-Related Outcomes

Ong et al. [28] investigated the impact of telehealth CBT-H on sleep inertia and sleep-problem-related quality of life. Their findings, however, indicated that no significant differences were observed at the 6-month follow-up time point.

### 4.6. Psychological Well-Being

Remarkably, despite the well-documented prevalence of anxiety and depression in narcolepsy patients [18,32] the available literature appears to be noticeably limited when it comes to evaluating the efficacy of psychological and behavioral interventions in managing these co-occurring conditions.

Ong et al. conducted a study to assess the feasibility and acceptability of a cognitive behavioral therapy protocol tailored to meet the needs of individuals with central disorders of hypersomnia (including narcolepsy) and comorbid depressive symptoms [28]. Participants were already receiving standard care, but as part of an additional non-pharmacological intervention, they received six weekly cognitive behavioral therapy sessions lasting about an hour each. These sessions were delivered via a videoconferencing platform, with participants assigned to individual or group formats. The findings revealed a significant reduction in depressive symptoms from baseline to post-treatment in 40% of the total sample. From baseline to post-treatment, 50% of participants who received group CBT-H experienced a significant reduction in depressive symptoms. Furthermore, effect sizes showed that group CBT-H had larger effects than individual CBT-H, implying that the group format may be more effective in addressing this outcome. Also, the intervention was effective in promoting self-efficacy (belief in one’s ability to carry out a behavior required to achieve a goal, even when the situation is unpredictable or stressful) in both individual and group settings. Self-efficacy is an important factor to consider, particularly when managing chronic health conditions in order to obtain better illness adaptation [33]. Exploratory analyses conducted across diagnostic groups (i.e., NT1, NT2, and idiopathic hypersomnia) indicated that the NT1 group reported clinically meaningful improvements in depression, anxiety, self-efficacy, social isolation, cognitive functioning, global mental health, and sleep inertia after the intervention. Only NT2 met the minimal clinically important difference benchmark on sleep disturbance and physical functioning.

Mundt et al. examined the development and implementation of a mindfulness-based intervention tailored for individuals with narcolepsy [27]. The intervention was adapted from the standard Mindfulness-Based Stress Reduction program to address specific challenges encountered by patients with narcolepsy, such as difficulties maintaining attention and alertness. Modifications included shorter session durations, a focus on practical strategies to manage narcolepsy symptoms (e.g., fatigue and hypersomnia), and a two-hour meditation retreat instead of the traditional full-day retreat. The intervention was delivered remotely via videoconferencing and offered in three formats: a brief four-week program emphasizing essential mindfulness principles with eight hours of instruction, a standard eight-week program with sixteen hours of instruction resembling the typical Mindfulness-Based Stress Reduction structure, and an extended twelve-week program with a more gradual learning pace and optional one-on-one support sessions. Participants were encouraged to engage in daily mindfulness practices lasting 30 to 45 min and to log their activities throughout the program. Assessments were conducted at baseline and post-treatment to evaluate changes in mindfulness, self-compassion, psychosocial functioning, mental health, and narcolepsy-specific symptoms. The study found that all three formats of the intervention were feasible and acceptable. Participants across all formats showed significant improvements in mindfulness and self-compassion, with moderate to large effect sizes. The extended program demonstrated the greatest clinical impact, meeting benchmarks for clinically meaningful improvements in anxiety, depression, cognitive and social functioning, self-efficacy, fatigue, and hypersomnia symptoms. The standard program also yielded substantial benefits, though fewer domains reached clinically significant thresholds compared to the extended program. The brief program, while less impactful, still demonstrated improvements in key outcomes such as emotional self-efficacy and fatigue.

### 4.7. Research Gap and Future Study Recommendations

Future research on psychological treatments for narcolepsy should address a number of important gaps in the current literature to advance our understanding and improve the quality of care for patients with this condition. While current research often focuses on symptom reduction, more studies are needed to explore the broader impact of behavioral and psychological treatments on the overall psychological well-being and quality of life of patients with narcolepsy. This includes assessing improvements in psychosocial functioning, emotional regulation, quality of life, and the ability to engage in meaningful daily activities.

Additionally, the current literature is disproportionately focused on adult populations, despite narcolepsy’s frequent onset during adolescence. Future studies should prioritize involving children and adolescents to understand the distinct developmental, social, and psychological challenges faced by younger individuals with narcolepsy. This emphasis will assist in developing interventions that are matched to the particular demands of these age groups and guide early intervention guidelines.

Future research should include longer follow-up periods to assess whether the observed benefits are maintained long-term, particularly in areas such as symptom management, psychological well-being, and patient adherence to behavioral strategies.

The main and secondary endpoints of the intervention (e.g., reducing daytime sleepiness, anxiety, and depression or improving coping mechanisms) should be clearly stated, and detailed descriptions of the methods and theories used to improve replicability should be provided.

Providers’ experience and qualifications in the provision of psychological interventions are oftentimes underreported. Information about providers’ professional background, such as clinical training, experience with sleep disorders, and specific expertise in the applied psychological intervention, should be reported.

The research presented in this review primarily focuses on sleep management and symptomatic aspects of psychological function among narcolepsy patients. While these aspects are critical, it is also worth noting that cognitive behavioral therapy interventions are frequently applied in chronic health conditions to promote healthy lifestyle behaviors [34]. These interventions are designed to address aspects such as diet and exercise that can be a valuable addition to the treatment of narcolepsy. Indeed, diet and regular exercise are critical in regulating the sleep–wake cycle and overall well-being [35,36]. Future research could explore the implementation of interventions aimed at promoting healthy lifestyle habits.

### 4.8. Limitations

Scoping reviews do not aim to assess the effectiveness of interventions but rather map the range of available literature. Therefore, our results do not allow for conclusions about the effectiveness of different psychological or behavioral interventions. Second, while we employed a comprehensive search strategy, we limited our review to peer-reviewed English-language studies, and this could have led to language and publication bias.

One intentional methodological decision of this review was to not impose a time constraint on the selection of included studies. We made this choice on the basis that there is not much psychological research on narcolepsy and that it is relatively new as an established condition. Although this choice broadened the scope of our review, we acknowledge that it may have introduced heterogeneity across study design, intervention models, and outcome measures. Future reviews can be enhanced by narrowing the timeframe or clearly defining the intervention criteria. This approach will facilitate a more homogeneous synthesis of findings and improve comparability among studies.

## 5. Conclusions

This review highlights the need for more research by indicating the lack of studies evaluating behavioral and psychological treatments in patients with narcolepsy. The significant heterogeneity in intervention methods, outcome measurements, and quality of methods limits conclusions. Despite these drawbacks, our review indicates that psychological interventions may be beneficial when supplemented with medication in the management of narcolepsy symptoms and psychopathology. The standardization of intervention procedures, extended follow-up periods, and more rigorous designs should be the main goals for future studies. Furthermore, studies need to be carried out to evaluate the effectiveness of these interventions in pediatric and adolescent populations.

## Figures and Tables

**Table 1:** Quality assessment scores according to the Crowe Critical Appraisal Tool (v1.4).

	Preliminaries (/5)	Introduction (/5)	Design (/5)	Sampling (/5)	Data Collection (/5)	Ethical Matters (/5)	Results (/5)	Discussion (/5)	Total (/40)
[25]	4/5	5/5	3/5	3/5	3/5	4/5	3/5	3/5	28 (70%)
[26]	4/5	4/5	4/5	3/5	4/5	NA	4/5	3/5	26/40 (65%)
[27]	5/5	5/5	4/5	4/5	4/5	5/5	4/5	5/5	36/40 (90%)
[28]	4/5	5/5	3/5	3/5	4/5	5/5	3/5	3/5	30/40 (75%)
[29]	4/5	5/5	4/4	3/5	4/5	3/5	4/5	3/5	30/40 (75%)
[30]	3/5	4/5	2/5	2/5	3/5	NA	3/5	3/5	20/40 (50%)

**Table 2:** Characteristics of the included studies.

Ref.	Country	Design	Study Aim	Sample Size (n, IG:CG)	Sex (n, Male: Female)	Age (yrs): Mean; SD; Range	Types of Diagnosis	Control Group (Type)	Follow-Up Points
[25]	Italy	Non-randomized control trial	To evaluate the efficacy of a meditation–relaxation intervention for SP in a group of patients with NT1 and NT2	10, 6:4	NR:NR	27.8; 12.2; NR	NT1 and NT2 + occurrence of SP at least four times during the last month	Deep breathing	8 weeks
[26]	USA	Pretest–posttest design	To reduce excessive daytime sleepiness	8	4:4	42.75; NR; 19–55	NT1	No CG	8 days
[27]	USA	Longitudinal study	To examine the acceptability and feasibility of a mindfulness-based intervention for narcolepsy, with three different program lengths, and the effect of the intervention on levels of mindfulness, self-compassion, psychosocial and neurocognitive functioning, and symptomatology	60	53:5 2: non-binary	35.6;12.2; NR	NT1 and NT2	No control group	4, 8, and 12 weeks
[28]	USA	Non-randomized trial	To determine the feasibility and acceptability of a novel cognitive behavioral therapy for depression daytime sleepiness, psychosocial functioning, and quality of life of people with CDH	35, 19 (IG1 individual): 16 (IG2 group)	3:32	32.0; 12.9; NR	CDH	No CG	6 weeks
[29]	USA	RCT	To determine whether an intervention combining scheduled sleep periods with stimulant medications was more effective in improving daytime sleepiness and severity of symptoms of patients with NT1 than the administration of stimulant medications alone	29, 9: 10 (CG1): 10 (CG2)	12:17	43.7; 13.9; 18–64	NT1	CG1 (two regularly scheduled 15 min naps per day); CG2 (regular sleep schedule for arising and retiring each day)	2 weeks
[30]	NR	Pretest–posttest design	To determine whether a specified number of scheduled naps would improve daytime alertness, reduce the severity of narcolepsy symptoms, and improve the quality of life of patients with NT1	16	7:9	46.8; 12.6; 21–65	NT1	No CG	4 weeks

Note. IG: intervention group; CG: control group; NT1: narcolepsy type 1; NT2: narcolepsy type 2; NR: not reported; CDH: central disorders of hypersomnia; RCT: randomized controlled trial.

**Table 3:** Outcomes and results of the included studies.

Ref.	Primary Outcomes (Measure)	Secondary Outcomes (Measure)	Drop-Out N (%)	Results (Primary Outcomes)	Results (Secondary Outcomes)
[25]	SP frequency in terms of the number of days; SP occurrence and the total number of SP episodes, duration of SP in the last month (SP-EPQ)	NR	NR	Significant reduction in the number of days in which SP occurred (*t*5 = 4.68, *p* = 0.002, one-tailed, *d* = 1.91) (IG: Δ = −5.50; CG: Δ = +0.25), as well as a 54% reduction in the total number of SP episodes (*t*5 = 3.86, *p* = 0.006, one-tailed, *d* = 1.57) (IG: Δ = −7.50; CG: Δ = +0.75). The reduction in episode duration was not significant (IG: Δ = −160.95; CG: Δ = +121.09).	NR
[26]	Excessive daytime sleepiness: descending subtraction test, a grammatical transformation or logical reasoning test, four-choice reaction time test, grip strength test, a measure of oral temperature, a number of additional subjective evaluation questions	NR	NR	The frequency of unscheduled sleep episodes did not exhibit a statistically significant difference across the various conditions; however, there was a marked reduction in the IG2 group compared to the IG1 group, approaching significance (*p* = 0.08). The number of unscheduled minutes of sleep recorded in the IG2 condition was less than that observed in the other conditions, approaching statistical significance when compared to IG1 (*p* = 0.07). Sleep efficiency was higher in the IG3 compared with the IG2 (IG3 > IG2 > IG1, *p* < 0.05). The percent of the scheduled bed period spent in active wakefulness was higher in IG2 compared to IG3 (IG2 > IG1 > IG3, *p* < 0.05). Reaction time significantly improved in IG2 compared to IG1 (IG2 > IG3 > IG1, *p* < 0.05). The grammatical transformation test results were lower in the napping condition than in the no-nap condition.	NR
[27]	Attendance, meditation practice, and data completeness	Mindfulness (FFMQ), self-compassion (SCS), mood (PROMIS), sleep (PROMIS, ESS, HSI, FOSQ) psychosocial functioning (PROMIS), and cognition (TMT, RBANS, SCWT, COWAT)	NR	Participants met the benchmarks for attendance, meditation, and data completeness in 71.7%, 61.7%, and 78.3% of cases, respectively. Moreover, a higher proportion of participants in the brief and extended intervention groups met these criteria compared to those in the standard group.	All intervention groups attained the minimal clinically important difference in mindfulness, self-compassion, emotional self-efficacy, positive psychosocial outcomes, overall mental health, and fatigue. Furthermore, both the standard and extended intervention groups achieved the minimal clinically important difference for reductions in anxiety and depression symptoms. The extended intervention group also exhibited clinically meaningful improvements in social and cognitive functioning, daytime sleepiness, hypersomnolence symptoms, and hypersomnia-related functional impairment.
[28]	Depression (PHQ), daytime sleepiness (ESS), psychosocial functioning (PROMIS)	Quality of life (FOSQ); sleep inertia (SIQ), sleep quality (RSQ)	8.5%	A total of 40% of the sample experienced a significant reduction in depressive symptoms from baseline to posttreatment (*p* < 0.0001, *d* = 0.80), and 50% of participants who underwent group-based CBT-H (IG2) reported symptom reduction. Effect size analyses from baseline to post-treatment suggested that IG2 demonstrated greater efficacy than the individual IG. The total sample increased in PROMIS self-efficacy from T0 to T1, with no significant differences between groups (*p* = 0.0009, *d* = 0.62). ESS decreased significantly at follow-up in the total sample, with no differences between groups (*p* = 0.04, *d* = −0.35).	Secondary outcomes did not change across follow-up points.
[29]	Excessive daytime sleepiness (24 h ambulatory polysomnographic monitoring) and severity of narcolepsy symptoms (NSSQ)	NR	NR	CG1 (scheduled naps) and CG2 (regular bedtimes) had almost identical reductions in daytime sleep (0.06 min; SE= 9.75 min), and IG (combination therapy) had much more reduction in daytime sleep duration than CGs (16.5 min; SE = 10.8 min). No differences were found between IG and CGs in NSSQ (*p* = 0.87). The effectiveness of scheduled sleep periods is strongly related to pre-treatment levels of daytime sleepiness. Participants with severe daytime sleepiness reported more benefit from the inclusion of scheduled sleep periods. On the contrary, participants with moderate or mild sleepiness did not (*p* = 0.028).	NR
[30]	Daytime alertness (as measured with the MWT), severity of narcolepsy symptoms (evaluated with the NSSQ), sleep attacks + time/duration of any naps + (evaluated with sleep diaries)	NR	NR	Mean sleep latency on the MWT increased significantly at the 4-week follow-up (*t*0: 7.4 ± 6.0 min; *t*1: 10.0 ± 5.8 min; *p* < 0.05. Sleep attacks did not change (*t*0: 0.5 ± 0.6 sleep attacks per day; *t*1: 0.6 ± 0.6), nor did any other symptoms.	NR

Note. (in alphabetical order): CG: control group; ESS: Epworth Sleepiness Scale; FFMQ: Five Facet Mindfulness Questionnaire; FOSQ: Functional Outcomes of Sleep Questionnaire; HSI: Hypersomnia Severity Index; IG: intervention group; MWT: Maintenance of Wakefulness Test; NR: not reported; PHQ: Patient Health Questionnaire; PROMIS: Patient-Reported Outcomes Measurement Information System measures; RSQ: Restorative Sleep Questionnaire; SIQ: Sleep Inertia Questionnaire; SP: sleep paralysis; SP-EPQ: Sleep Paralysis Experiences and Phenomenology Questionnaire; SCS: Self-Compassion Scale; SCWT: Stroop Color and Word Test; TMT: Trail Making Test; RBANS: Repeatable Battery for the Assessment of Neuropsychological Status; COWAT: Controlled Oral Word Association Test.

**Table 4:** Characteristics of the intervention.

Ref.	Setting	Provider (Background)	Duration of Intervention (Number of Sessions)	Clinical Approach	Intervention Approach	Format	Brief Description of the Intervention
[25]	At home	Experimenter (NR)	8 weeks (NR)	Cognitive behavioral therapy	Psychoeducational + psycho-behavioral strategies	In-person or video call instruction + at-home implementation	Meditation and relaxation therapy is an intervention for sleep paralysis which includes the use of the following strategies during the sleep paralysis attack: step I: reappraisal of the meaning of the attack; step II: psychological and emotional distancing; step III: inward focused-attention meditation; step IV: muscle relaxation.
[26]	In a bed-and-breakfast establishment in a quiet rural setting	NR	8 days (8)	Behavioral therapy	Behavioral	In-person instruction + at-home implementation	Following a two-day adaptation phase during which participants were allowed to sleep ad libitum, three experimental sleep protocols were implemented, each ensuring an equivalent total sleep duration within a 24 h period. In the first condition (IG1), sleep was consolidated into a single nocturnal episode with no daytime naps. The second condition (IG2) employed a long nap protocol, while the third condition (IG3) utilized a multiple short nap protocol. Both IG2 and IG3 involved a reduction in nocturnal sleep, which was supplemented by daytime sleep either through a single extended nap (IG2) or five evenly distributed short naps (IG3) during the remaining wakefulness period.
[27]	At home	Mindfulness Instructor	Brief Mindfulness-based intervention: 4 weeks (4) Standard mindfulness-based intervention: 8 weeks (8) Extended mindfulness-based intervention: 12 weeks (12)	Mindfulness	Psychoeducational– experiential	Online intervention	The intervention was based on the content and structure of mindfulness-based stress reduction. During each session, the instructor offered educational guidance, facilitated group discussions, and guided participants through mindfulness exercises such as body scan meditation, seated meditation, walking meditation, and yoga.
[28]	At home	4 therapists (2 therapists were licensed clinical psychologists, 1 therapist was a postdoctoral fellow, and 1 therapist was a doctoral student in clinical psychology)	6 weeks (6 sessions of 1 h each)	Cognitive behavioral therapy for hypersomnia	Psychoeducational + psycho-behavioral strategies	Online intervention	CBT-H was designed as a modular treatment to address issues related to quality of life for both narcolepsy and idiopathic hypersomnia. Each module consisted of a specific psychological and/or behavioral activities and homework assignments that were customized to provide flexibility in addressing disease-specific symptoms of people with NT1, NT2, and idiopathic hypersomnia.
[29]	At home	NR	2 weeks (NR)	Behavioral therapy	Behavioral	In-person instruction + at-home implementation	Scheduled naps combined with regular bedtimes.
[30]	At home	Polysomnographic technologist	4 weeks (NR)	Behavioral therapy	Behavioral	In-person instruction + at-home implementation	Three regularly scheduled naps.

Note. CBT-H: cognitive behavioral therapy for hypersomnia; IG: intervention group; NT1: narcolepsy type 1; NT2: narcolepsy type 2; NR: not reported.

## Data Availability

Not applicable.
